# Relevance of laboratory testing for the diagnosis of primary immunodeficiencies: a review of case-based examples of selected immunodeficiencies

**DOI:** 10.1186/1476-7961-9-6

**Published:** 2011-04-09

**Authors:** Roshini S Abraham

**Affiliations:** 1 Department of Laboratory Medicine and Pathology, Mayo Clinic, Rochester, MN, USA

## Abstract

The field of primary immunodeficiencies (PIDs) is one of several in the area of clinical immunology that has not been static, but rather has shown exponential growth due to enhanced physician, scientist and patient education and awareness, leading to identification of new diseases, new molecular diagnoses of existing clinical phenotypes, broadening of the spectrum of clinical and phenotypic presentations associated with a single or related gene defects, increased bioinformatics resources, and utilization of advanced diagnostic technology and methodology for disease diagnosis and management resulting in improved outcomes and survival. There are currently over 200 PIDs with at least 170 associated genetic defects identified, with several of these being reported in recent years. The enormous clinical and immunological heterogeneity in the PIDs makes diagnosis challenging, but there is no doubt that early and accurate diagnosis facilitates prompt intervention leading to decreased morbidity and mortality. Diagnosis of PIDs often requires correlation of data obtained from clinical and radiological findings with laboratory immunological analyses and genetic testing. The field of laboratory diagnostic immunology is also rapidly burgeoning, both in terms of novel technologies and applications, and knowledge of human immunology. Over the years, the classification of PIDs has been primarily based on the immunological defect(s) ("immunophenotype") with the relatively recent addition of genotype, though there are clinical classifications as well. There can be substantial overlap in terms of the broad immunophenotype and clinical features between PIDs, and therefore, it is relevant to refine, at a cellular and molecular level, unique immunological defects that allow for a specific and accurate diagnosis. The diagnostic testing armamentarium for PID includes flow cytometry - phenotyping and functional, cellular and molecular assays, protein analysis, and mutation identification by gene sequencing. The complexity and diversity of the laboratory diagnosis of PIDs necessitates many of the above-mentioned tests being performed in highly specialized reference laboratories. Despite these restrictions, there remains an urgent need for improved standardization and optimization of phenotypic and functional flow cytometry and protein-specific assays. A key component in the interpretation of immunological assays is the comparison of patient data to that obtained in a statistically-robust manner from age and gender-matched healthy donors. This review highlights a few of the laboratory assays available for the diagnostic work-up of broad categories of PIDs, based on immunophenotyping, followed by examples of disease-specific testing.

## Introduction and Outline

Since the topic of primary immunodeficiencies (PIDs) and the associated diagnostic testing is exhaustive and highly complex [1], this review article will focus primarily on 2 key methodologies used for the laboratory diagnosis of PIDs - flow cytometry and genetic testing, by offering case-based examples.

The hallmark of most PIDs is susceptibility to recurrent and life-threatening infections, since the cardinal role of the immune system is host defense. However, the clinical spectrum of PIDs is very diverse and can include other manifestations such as autoimmunity, neoplasia, and congenital anomalies of organs and/or skeleton. Therefore, the traditional role of the laboratory has been to provide supportive data to a largely clinical, radiological and family history-based diagnostic approach. The development of reagents capable of identifying disease-specific mutated proteins along with the ability to evaluate multiple subsets of immune cells and their function, such as respiratory burst, proliferation or phosphorylation, simultaneously, facilitated the incorporation of multi-color and functional flow cytometry into the diagnostic work-up for PIDs.

While flow cytometry may be diagnostic for many PIDs where specific proteins and/or defective function can be directly assessed (Table 1)[2-4], the relevance of confirming the diagnosis by genetic testing or mutation analysis still remains germane,[5,6] especially when protein is present but non-functional. Further, genetic testing can provide a venue for genetic counseling by aiding in the identification of carriers, particularly for X-linked diseases, as well as enabling prenatal diagnosis. It is particularly helpful in elucidating the correlation between phenotype and genotype, when there are either allelic variants or unusual presentations present, leading to prognostic insights. But, surpassing all these is the role of genetic testing in identifying asymptomatic individuals who carry a defective gene associated with a potentially lethal PID, prior to clinical and/or other immunological manifestations of disease, facilitating early therapeutic intervention, and this is exemplified by the newborn screening program for severe combined immunodeficiencies (SCID) and T cell lymphopenia (discussed later in this review). The enaction of federal legislation (GINA 2008, Genetic Information Nondiscrimination Act) now protects patients who obtain genetic testing from any form of financial, health or other discrimination, facilitating implementation of diagnostic genetic testing when appropriate[7].

**Table 1:** List of only those PIDs where screening diagnosis can be made by specific protein detection by flow cytometry

PID	Disease-specific protein detected by flow*
X-linked agammaglobulinemia (XLA)	Bruton's tyrosine kinase (Btk) in monocytes, platelets
Wiskott-Aldrich syndrome (WAS) and related allelic variants, X-linked thrombocytopenia (XLT) and X-linked neutropenia/myelodysplasia	Wiskott-Aldrich Syndrome protein (WASP)
X-linked Hyper IgM syndrome (XL-HIGM)	CD40L (CD154) on activated T cells
Hyper IgM syndrome type 3	CD40 on B cells and/or monocytes
CVID-associated defects	ICOS (activated T cells), CD19, BAFF-R, TACI
Familial Hemophagocytic Lymphohistiocytosis (fHLH)	Perforin in NK cells and CD8 T cells
X-linked lymphoproliferative disease (XLP)	SAP (SH2D1A)
X-linked inhibitor of apoptosis (XLP2) disease	XIAP (BIRC4)
Chronic Granulomatous disease (CGD) - Autosomal recessive	p47phox, p67phox, p22phox in neutrophils
Leukocyte Adhesion deficiency type 1 (LAD-1)	CD18, CD11a, CD11b on leukocytes
Leukocyte Adhesion deficiency type 2 (LAD-2)	CD15 (Sialyl-Lewis ^X^) on neutrophils and monocytes
Interferon gamma receptor 1 deficiency	IFNγR1
Interferon gamma receptor 2 deficiency	IFNγR2
IL-12 and IL-23 receptor β1 deficiency	IL-12Rβ1
STAT1 deficiency	pSTAT1
STAT5B deficiency	pSTAT5
Immunodeficiency, enteropathy, X-linked (IPEX)	FOXP3 on regulatory T cells (Tregs, CD4+CD25+FOXP3+)
Warts, Hypogammaglobulinemia, and myelokathexis (WHIM)	CXCR4 on T cells
Common gamma chain (cγ chain)	CD132 (IL-2RG, IL-4RG, IL-7RG, IL-9RG, IL-15RG) on activated T cells
Bare Lymphocyte Syndrome type I and II (BLS I and II)	MHC class I and II expression on monocytes, B cells and T cells (activated) respectively
CD25 deficiency (IPEX-like syndrome)	CD25 (IL2Rα)
Membrane cofactor protein (MCP) deficiency	CD46
Membrane attack complex deficiency (MAC)	CD59

* Presence of protein as detected by flow cytometry does not rule out an underlying functional mutation, therefore, results have to be correlated with other laboratory and immunological parameters, including functional flow cytometry when applicable, clinical and family history and confirmed by genetic testing for final diagnosis. Details of these individual defects can be found in "Immunologic Disorders in Infants and Children, 5^th ^Ed, Eds. R. Stiehm, H. Ochs and J. Winkelstein, 2005, Elsevier Saunders).

The classification of PIDs has been primarily based on the chief component(s) of the immune system affected resulting in at least 8 broad categories - combined T and B cell, predominant antibody, well-defined PIDs, immune dysregulation, phagocyte-associated, innate immunity, autoinflammatory, and complement defects[8]. But, these categories are by no means exclusive and there can be considerable clinical and immunological overlap between them. There are other approaches to classification[9], which can include immunophenotyping for specific PIDs, as will be discussed later in this review.

To limit the scope of this review, the following PIDs will be used as examples for the laboratory diagnostic work-up: X-linked agammaglobulinemia (XLA), Chronic Granulomatous Disease (CGD), and Wiskott - Aldrich syndrome (WAS)/X-linked thrombocytopenia (XLT).

## Case 1

A 51 year old male presents to an adult immunodeficiency clinic for evaluation of a life-long history of recurrent sinopulmonary infections. Diagnostic work-up done elsewhere at a prior evaluation revealed profound hypogammaglobulinemia (IgG, IgA and IgM) for which he was initiated on intravenous immunoglobulin (IVIG) at the age of 28 years, but he was never given a clear diagnosis of the underlying medical problem. On his recent visit to the above-mentioned immunodeficiency clinic, an immunologic assessment was performed, which included lymphocyte subset quantitation, immunoglobulin levels along with documentation of clinical history. Not surprisingly, the IgG levels were within normal range (due to the IVIG) but the IgA and IgM were undetectable. The flow cytometric quantitation of T, B and NK cells were significant for an almost complete absence of CD19+ (and CD20+) B cells (0%, 2 cells/uL). No pertinent family history was obtained from the patient and the patient was given a diagnosis of Common Variable Immunodeficiency (CVID). Management of the patient was essentially unchanged since the patient was already receiving replacement immunoglobulin therapy, and prophylactic versus therapeutic use of antibiotics was discussed.

The case was referred to a laboratory immunologist to determine if the diagnosis of CVID was indeed accurate for this patient. Based on the clinical history of life-long recurrent infections, male gender, very low levels of immunoglobulins and nearly absent B cells, the differential diagnosis should have also included X-linked agammaglobulinemia (XLA), despite the age of the patient (5^th ^decade of life).

**Figure 1:**
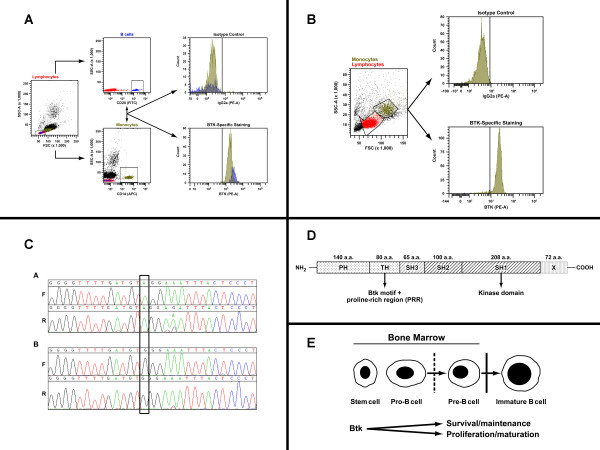
**Evaluation for X-linked agammaglobulinemia (XLA)**. A) Flow cytometric evaluation for Btk protein in a healthy control. B) Flow cytometric evaluation for Btk protein in Case 1 patient. C) Full-gene sequencing in the *BTK *gene for mutation analysis in Case 1 patient. D) Schematic representation of Btk protein structural organization. E) Schematic representation of Btk in B cell development.

Laboratory testing was undertaken to evaluate for Bruton's tyrosine kinase (Btk) protein, typically present intracellularly in monocytes, B cells and platelets. Intracellular flow cytometry was performed on B cells and monocytes of a healthy control and monocytes from the patient (since B cells were absent) (Figure 1A and 1B). The analysis revealed normal expression of Btk protein within the monocytes from the patient. However, since certain mutations can permit protein expression while abrogating function, it is important to follow protein analysis with genotyping. Full-gene sequencing (which refers to the sequencing of the entire coding region of the gene with intron-exon boundaries and the 5' and 3' untranslated regions -UTRs) revealed a nonsense mutation, W588X in exon 18 (old nomenclature; exon 17 - new nomenclature since the first exon of the *BTK *gene is non-coding) of the *BTK *gene, which contributes to the kinase domain in the protein (Figure 1C). This mutation resulted in premature truncation of the protein (loss of 72 amino acids from the 3' end of the kinase domain), which permitted intracellular protein expression but affection function of the protein (Figure 1D).

This additional laboratory analysis allowed a correct diagnosis of XLA to be provided to this patient, which in this case did not change medical management (use of IVIG) but provided a venue for discussing the significance of monogenic defects, such as XLA and appropriate genetic counseling for at-risk family members, such as carrier offspring.

To date, a total of 7 patients, including this patient have been identified as having this particular mutation within the *BTK *gene.

The *BTK *gene has 19 exons, 18 of which are coding and to date, over 600 mutations have been described within this gene as being associated with the clinical phenotype of XLA.

XLA is a primary B-cell deficiency [10] characterized by recurrent respiratory or gastrointestinal tract infections, usually within the first year of life, though the above case exemplifies that a diagnosis may not be made till much later in adult life, even if appropriate treatment is empirically initiated based on infectious history, immunoglobulin levels and absence of vaccine-specific antibody responses. Besides the hypogammaglobulinemia, absence or dramatic reduction in the number of circulating B cells is another hallmark of this disease, because the Btk protein is critical for B cell development within the bone marrow and maturation in the periphery (Figure 1E).

XLA can often be misdiagnosed as CVID in adults because of overlapping features, such as hypogammaglobulinemia and recurrent infections. However, only 5% of CVID cases have less than 1% of peripheral CD19+ B cells [11]. Hypoplasia of secondary lymphoid tissue, such as tonsils, adenoids and lymph nodes can be helpful in adults to confirm a presumptive diagnosis, however, this feature is not useful in newborns and very young infants as the hypoplasia may not be apparent due to the lack of antigen-driven expansion of B cells at that age.

Therefore, XLA should be in the differential diagnosis of a male patient who presents with recurrent sino-pulmonary infections, profound hypogammaglobulinemia of the 3 major isotypes, absent or decreased peripheral B cells, neutropenia, Giardia-associated diarrhea, sepsis, meningitis or encephalitis with absent or hypoplastic lymphoid structures. The susceptibility of XLA patients to bacterial and enteroviral (single-stranded RNA viruses) infections may be related to defective Toll-like receptor (TLR) signaling in dendritic cells (DCs) in patients with XLA[12,13], though TLR signaling and downstream effector functions in neutrophils have been shown to be normal[14].

There can be considerable phenotypic heterogeneity including age of presentation depending on the nature and location of the mutation within the gene[15]. In a study of 201 US patients with XLA, it was determined that infection was the dominant clinical presentation, though in a small proportion of patients, family history was the initial presentation. A quarter of these patients had both infection and family history, and smaller numbers also had neutropenia [16]. The diagnostic criteria included a positive family history, absent B cells and hypogammaglobulinemia and identification of mutations within the *BTK *gene [16].

Laboratory testing is available in larger reference laboratories for flow cytometric-based evaluation of Btk protein [17,18] and full-gene or known mutation sequencing. It is critical to perform a complete evaluation, including genetic testing since there is a large spectrum of variability in the phenotype depending on the nature of the specific *BTK *mutation [15,19,20] and this would be relevant for future genetic testing and counseling as well as genotype-phenotype correlations.

For genetic counseling purposes, if a female individual has one affected male child and any other affected male relative, then she should be regarded as an obligate carrier. Approximately half (50%) of male XLA patients do not have family history of the disease, and therefore, either have a *de novo *or spontaneous mutation (~15-20% of patients) or the mother is a carrier of the mutation (majority of cases, 80-85%). All the female offspring of an affected male patient will be obligate carriers of the mutation. While carrier females for X-linked diseases can usually be identified by flow cytometry due to random X-chromosome inactivation resulting in two populations for the protein being tested, there are some individuals who can be missed when the specific mutation permits Btk protein expression, and therefore, genetic testing is the most robust method for identifying carriers. Typically, the familial disease-causing mutation should be known for carrier genetic testing for at-risk female relatives, or asymptomatic male infants of carrier females, and for prenatal diagnostic testing. It is possible to perform full-gene sequencing in carriers if the specific disease-causing mutation is not known, however, if a novel mutation is identified in the female carrier, it would require clinico-pathological correlation and identification of the same mutation in affected male relatives to establish its clinical significance. Prenatal diagnosis in a male fetus (46, XY) requires prior knowledge of the disease-causing mutation.

## Case 2

A 46 year old male presented to the Nephrology Clinic within a large Transplant Center for evaluation related to the need for a third renal transplant. His prior history was significant for bloody, persistent diarrhea in childhood and he was later on shown to have thrombocytopenia. He also had a history of eczema in childhood, which resolved over time. His childhood and early adulthood was otherwise uneventful with no significant bleeding history, but there was occasional minor bruising. The history was notable for lack of recurrent infections in childhood or early adult life. Twelve years prior to this presentation, he was found to have evidence of chronic renal disease, secondary to glomerulonephritis and as a result also developed hypertension. Three years following the discovery of chronic renal failure, he received a living related donor renal transplant with no evidence of acute rejection episodes. However, two years post-transplant, there was pathologic and clinical evidence of chronic allograft nephropathy with BK viremia, indicating likely BK virus (BKV)-associated nephropathy.

Two years following the identification of BK nephropathy, he received a second living related donor transplant, again with no acute rejection episodes. But, one year following the 2^nd ^transplant, there was evidence of BK nephropathy again with BK viremia, for which he was treated with Lefluonomide and Cidofovir. The maintenance immunosuppression for the transplant was Rapamycin. He was evaluated again five years after the 2^nd ^transplant for worsening renal function. Laboratory evaluation revealed lymphopenia with a total CD45 lymphocyte count of 0.77 (see Table 2 for reference values for key lymphocyte subsets), CD3 T cells = 491 cells/uL, CD4 = 238 cells/uL, CD8 = 240 cells/uL, CD19 B cells = 60 cells/uL and NK cells == 208 cells/uL, CD4:CD8 ratio = 0.99. There was both CD4+ T cell and CD19+ B cell lymphopenia present. Further analysis of B cell subsets revealed decreased class-switched memory B cells (CD19+CD27+IgM-IgD-) and marginal zone B cells (CD19+CD27+IgM+IgD+). Immunoglobulin levels were normal (IgG = 685, IgA = 228 and IgM = 48 mg/dL). BK viremia was significant with 11500 copies/ml and BK viruria was at 3465000 copies/ml.

**Table 2:** Normal reference values for lymphocyte subsets in healthy adults determined by flow cytometry

Lymphocyte subset	95% reference values	
	*18-55 years*	*>55 years*
CD45	0.99 - 3.15 thousand/uL	1.00 - 3.33 thousand/uL
CD3	677-2383 cells/μl	617-2254 cells/μl
CD4	424-1509 cells/μl	430-1513 cells/μl
CD8	169-955 cells/μl	101-839 cells/μl
CD19	99-527 cells/μl	31-409 cells/μl
CD16+56+	101-678 cells/μl	110-657 cells/μl
		
CD3	59-83%	49-87%
CD4	31-59%	32-67%
CD8	12-38%	8-40%
CD19	6-22%	3-20%
CD16+56+	6-27%	6-35%

Data derived from 207 healthy adult male and female donors. Pediatric reference ranges for T, B and NK cells [147].

The early childhood history of bloody diarrhea and thrombocytopenia without recurrent infections raised the diagnostic suspicion of a mild phenotype of Wiskott-Aldrich syndrome (WAS) or the related X-linked thrombocytopenia (XLT). Flow cytometric evaluation of intracellular WAS protein [21,22] revealed 67% positive lymphocytes for WASP (moderate intensity staining), 83% positive granulocytes and 92% positive monocytes (though staining intensity on the latter 2 populations was dim; reference range for % positive WASP populations = 95-100%).

**Figure 2:**
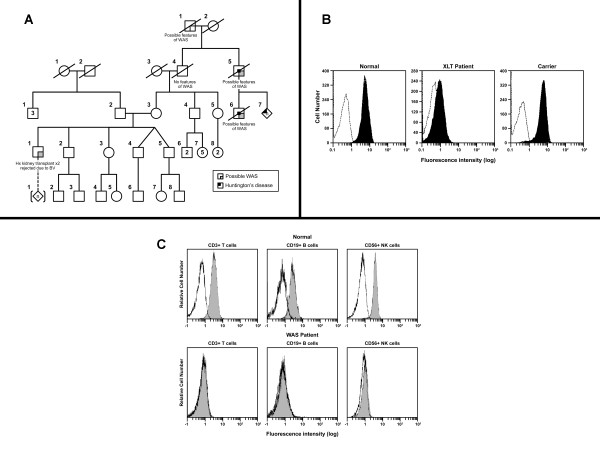
**Evaluation for Wiskott-Aldrich syndrome (WAS) and related allelic variant, X-linked thrombocytopenia (XLT)**. A) Pedigree analysis for patient (Case 2) with X-linked thrombocytopenia (XLT). B) Flow cytometric analysis for Wiskott-Aldrich syndrome protein (WASP) in lymphocytes in XLT patient and carrier. Figure reproduced with permission of American Society of Hematology, from "X-linked thrombocytopenia identified by flow cytometric demonstration of defective Wiskott-Aldrich syndrome protein in lymphocytes", Kanegane et al, 95: 1110-1111, 2000; permission conveyed through Copyright Clearance Center, Inc [38]. C) Flow cytometric analysis for Wiskott-Aldrich syndrome protein (WASP) in lymphocytes in WAS patient. Figure reprinted from Journal of Immunological Methods, 260, Kawai et al., Flow cytometric determination of intracytoplasmic Wiskott-Aldrich syndrome protein in peripheral blood lymphocyte subpopulations, p.195-205 [21], Copyright (2000), with permission from Elsevier.

To confirm the flow cytometric findings and identify the specific disease variant in this patient, full-gene sequencing (including intron-exon boundaries) of the WAS gene was performed, and revealed a splice-site mutation in intron 6 (IVS 6+5, 559+5; G>A), which resulted in a frameshift mutation with a premature termination of the protein at 190 amino acid residues (502 amino acids for the full-length protein). Other reports have shown that this mutation is associated with XLT, an allelic variant of WAS [23], and is in fact a "hotspot" mutation found in approximately 9% of patients with XLT [24]. The genetic pedigree of the patient (Figure 2A) did not reveal a clear or well-documented family history of WAS or XLT though there were relatives with possible features of WAS/XLT.

WAS is an X-linked disease characterized by a clinical triad of thrombocytopenia, eczema and recurrent infections, but these features may be seen in only 1 out of 4 WAS patients so the initial diagnosis can be easily overlooked. The most reliable features of WAS are thrombocytopenia (platelet count less than 70,000 in a patient without splenectomy) with low platelet volume (<5fl) [25,26]. Approximately 1/3^rd ^of WAS patients have a life-threatening bleeding episode prior to diagnosis. Recurrent sino-pulmonary infections as well as viral infections (Varicella, HSV 1 and 2, molluscum contagiosum, and warts) are common. Eczema is seen in the majority of WAS patients (>80%) while eosinophilia is seen in greater than 30% of patients and elevations in IgE levels are not uncommon. Autoimmune and inflammatory manifestations are quite common (approximately 40-72% of patients) and about a quarter of these patients have multiple autoimmune features. Autoimmune hemolytic anemia (AIHA) is the most common autoimmunity seen in WAS patients (~36%) and is a poor prognostic factor.

Profound immunological anomalies are present in WAS patients and include defects in both cellular and humoral immunity. While lymphopenia can develop over time, typically IgG levels are normal with normal to low IgM, and increased IgA and IgE. There is evidence of decreased class-switched memory B cells and antibody responses to vaccine antigens, both protein and polysaccharide, are low, while responses to live viral antigens are paradoxically normal. Lymphocyte proliferative responses to mitogens, antigens and anti-CD3 stimulation are low. NK cell function and leukocyte chemotaxis are variable, and most, but not all WAS patients have low CD43 (sialophorin) expression on T cells [25-27].

Mutations in WAS are associated with distinct clinical phenotypes, and mutations that significantly affect WAS protein function lead to the most severe phenotype, which is further complicated by autoimmunity and malignancies [25,28]. XLT is an allelic variant of WAS [29-32] and is characterized by thrombocytopenia and small platelets. Typically, serious immunological anomalies are uncommon in XLT, though elevated IgA and IgE and mild eczema can be present. XLT patients have a higher risk of sepsis after splenectomy and slightly higher risk for neoplasia, autoimmunity and IgA nephropathy [24,33,34]. Missense mutations in exon 1 and 2 of the WAS gene are most commonly associated with XLT, in fact, 3/4^ths ^of the mutations in XLT are missense and approximately 12% are splice-site [23,31]. Other allelic disease variants due to WAS mutations include intermittent thrombocytopenia [35] and congenital X-linked neutropenia without the clinical characteristics of WAS or XLT [36,37]. Somatic reversions have been reported in several WAS patients where the disease-causing mutation has spontaneously reverted to wild-type state in subsets of hematopoietic cells resulting in somatic mosaicism [25].

While WAS and XLT in male patients and female carriers can be identified in the laboratory by flow cytometric analysis as previously mentioned (Figure 2B and 2C) [38,39], the role of genetic testing cannot be understated due to the above-described allelic variants, which highlight the genotype-phenotype variability observed in this immunodeficiency.

Returning to the patient presented here, it is quite evident from the clinical history, flow cytometric evaluation of WAS protein (WASP) and WAS gene sequencing that the patient has a diagnosis of XLT. His renal disease was likely related to the underlying WAS mutation since WAS variants with increased IgA and impaired renal function have been reported [40], but his recurrent BKV infection and associated nephropathy suggest impaired immunological function, related to the XLT, which coupled with transplant immunosuppression is likely responsible for a profound immune compromise, and recurrent loss of allografts. Therefore, in patients with XLT or WAS undergoing renal transplantation, it may be worthwhile re-thinking conventional immunosuppression approaches due to the underlying immunodeficiency. Also, knowing the specific genetic diagnosis provides helpful information on additional screening for the patient due to the increased risk of malignancy [34].

It should also be kept in mind that female carriers of X-linked diseases can be clinically symptomatic if there is skewing of lyonization and resultant inactivation of the wild-type X-chromosome, as has been reported for XLT [41], XLA [42], and X-linked CGD [43-46].

## Cases 3 and 4

A 19 year old male presented to an immunodeficiency practice with a history of peri-rectal fistulas at 7 years of age, followed by a deep left neck abscess refractory to antibiotics at 10 years of age. In general, he had a history of at least 1 skin infection per year. The causal microbe was usually methicillin-sensitive *Staphylococcus aureus *(MSSA) with no evidence of *Aspergillus*, *Nocardia*, *Pseudomonas *or *Serratia *species. At presentation in the recent visit he reported a peri-rectal abscess one month prior and bloody diarrhea for 1 week with sharp, diffuse abdominal pain, nausea and vomiting, fever, chills and a weight loss of 12 lbs. He was unresponsive to high-dose steroids. His laboratory data revealed both IgA and IgG antibodies to *Saccharomyces cerevisiae*, no evidence of *Clostridium difficile *and the stool culture was also negative for any pathogenic organisms but positive for leukocytes. Colonoscopy showed abnormal wall thickening of all segments of the colon and rectum. A diagnosis of severe colitis and perianal fistula was initially provided, and the rectal biopsy revealed moderate colitis with acute cryptitis and focal abscess formation. The childhood history of fistulas and abscesses with *Staphylococcus *raised concerns for Chronic Granulomatous Disease (CGD).

**Figure 3:**
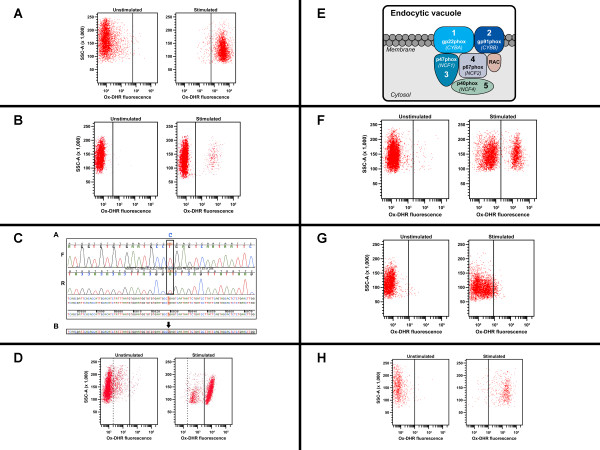
**Evaluation for Chronic Granulomatous Disease (CGD)**. A) Flow cytometric analysis for neutrophil oxidative burst (NOXB) in a healthy control. B) Flow cytometric analysis for neutrophil oxidative burst (NOXB) in a patient with X-linked Chronic Granulomatous Disease (XL-CGD), Case #3. C) Full-gene sequencing in the *CYBB *gene for mutation analysis in Case 3 patient. D) Flow cytometric analysis for neutrophil oxidative burst (NOXB) in mother of patient with X-linked Chronic Granulomatous Disease (XL-CGD), Case #3. E) Schematic representation of NADPH oxidase. F) Flow cytometric analysis for neutrophil oxidative burst (NOXB) in a carrier with X-linked Chronic Granulomatous Disease (XL-CGD), Case #4. G) Flow cytometric analysis for neutrophil oxidative burst (NOXB) in a patient with autosomal recessive CGD (AR-CGD). H) Flow cytometric analysis for neutrophil oxidative burst (NOXB) in a carrier with autosomal recessive CGD (AR-CGD).

Laboratory evaluation was performed for neutrophil oxidative burst using dihydrorhodamine (DHR) flow cytometry before and after stimulation of neutrophils with Phorbol Myristate Acetate (PMA) (Figure 3A - normal, healthy donor and 3B - patient). There was no evidence of DHR fluorescence after stimulation in the majority of the neutrophils (96%) consistent with a phenotype observed in X-linked CGD (XL-CGD) (Figure 3B). However, it was interesting to note that 4% were positive for modest levels of DHR fluorescence after stimulation, which may be suggestive of somatic mosaicism due to spontaneous reversion in a subset of neutrophils. Genetic testing was performed with full-gene sequencing and revealed a nonsense mutation (R130X) in exon 5 of the *CYBB *gene, which encodes the gp91phox protein (Figure 3C). This result along with the flow cytometry data was consistent with a diagnosis of XL-CGD. Flow cytometric analysis (Figure 3D) and genetic testing (data not shown) was performed on the mother of the patient and revealed that she was not a carrier of the disease-causing mutation, and therefore, the patient had a *de novo *or spontaneous mutation that accounted for his clinical phenotype of CGD.

A second patient, a 23 year-old female was seen in the same immunodeficiency clinic as the above-mentioned male patient. The female patient was diagnosed with Crohn's disease at the age of 13 years when she had abdominal pain, fatigue and hematochezia. She underwent exploratory endoscopy and colonoscopy and her biopsy showed evidence of mild to active small bowel and colonic colitis with non-necrotizing granulomas. Her prior history was significant for skin abscesses, at least once per year, on the upper arm, gluteal region, thighs, vulvar and vaginal areas. There was no evidence of pneumonia, sinusitis, osteomyelitis, cellulitis or meningitis. She was treated almost continuously with immunosuppressive and biological therapies along with steroids since the initial diagnosis of Crohn's disease. Her family history was remarkable for XL-CGD and ocular complications of CGD. Flow cytometric testing for neutrophil oxidative burst revealed 2 populations for DHR fluorescence with a larger negative and smaller positive population (Figure 3E). Genetic testing revealed a heterozygous deletion of 16 nucleotides (c.360-375del16). The patient's mother and two maternal aunts carried the same deletion mutation (one of these maternal aunts also had ulcerative colitis and primary biliary cirrhosis), and one maternal uncle died at the age of 18 months with recurrent neck abscesses. The family history also revealed two maternal great-uncles who died in childhood of unknown causes, but presumed CGD.

The clinical history of inflammatory bowel disease (IBD), recurrent skin abscesses (facial, labial, peri-rectal), poor surgical wound healing, aphthous ulcers and ocular complications all suggest a clinical phenotype of XL-CGD, due to skewing of X-chromosome inactivation (lyonization). The DHR flow cytometry results indicate that there at least 30% neutrophils with normal oxidative burst function. Similar analyses done elsewhere showed positive DHR populations between 19-26%. It has been reported that if there are greater than 10% of neutrophils with normal oxidative burst, there is typically no evidence of a clinical phenotype [47-50].

CGD is a relatively rare primary immunodeficiency with an incidence of approximately 1 in 200,000 to 250,000 individuals characterized by defects in the oxidative burst pathway that is linked with phagocytosis in myeloid cells, such as neutrophils. The primary defect in CGD is associated with the key enzyme involved in generation of the respiratory burst, NADPH oxidase. This enzyme has at least 5 subunits (Figure 3F), two of which are membrane-bound, gp91phox (*CYBB *gene) and gp22phox (*CYBA *gene), and three are cytosolic components, p47phox (*NCF1 *gene), p67phox (*NCF2 *gene) and p40phox (*NCF4 *gene). The p40phox primarily interacts with p67phox and forms a larger complex with p47phox, which in turn interacts with a RacGTPase, *RAC1*, permitting translocation to the membrane upon stimulation where it activates the catalytic core of the NADPH oxidase formed by the gp91phox and p22phox proteins. The most common form of CGD is X-linked accounting for approximately 70% of cases, due to mutations in the *CYBB *gene. The remaining 30% of cases are associated with mutations in the other subunits and inherited in an autosomal recessive (AR) manner. Mutations in *NCF1 *account for ~25% of the AR cases, while *NCF2 *and *CYBA *mutations are quite rare. The most recent NADPH subunit in which mutations were found to be associated with CGD was the p40phox (*NCF4*) reported in a single patient [51].

Clinically, CGD is characterized by recurrent bacterial and fungal infections of primarily the lungs, gastrointestinal tract, skin, and lymph nodes [52] caused largely by a relatively small number of pathogens - *Staphylococcus aureus*, *Aspergillus *species, *Serratia marcescens*, *Salmonella *species, *Burkholderia *(*Pseudomonas*) *cepacia*. Most of these pathogens are catalase-positive organisms. The most common clinical manifestations are pneumonia, cutaneous abscesses, lymphadenitis and chronic inflammatory reactions resulting in granulomas.

Carriers of XL-CGD and AR-CGD are usually asymptomatic, however, about 50% of XL-carriers have been reported to have recurrent mouth lesions, manifesting as either gingivitis or stomatitis. Further, skewing of X-chromosome inactivation (lyonization) with inactivation of the normal X-chromosome has been reported in CGD, which could potentially confer a mild clinical phenotype in the female carrier, though this typically does not happen until the proportion of skewed, inactivated neutrophils drops below 10%, as stated previously, [47-50], though healthy carriers with less than 10% normal neutrophils have also been reported [53]. The female carrier for XL-CGD presented in this article had, at all the time-points tested, greater than 10% neutrophils that were positive for oxidative burst, yet there was evidence of a clinical phenotype with recurrent skin infections and the IBD-like colitis. Further, age-related changes in X-chromosome inactivation patterns have been shown to change the relative proportion of normal to abnormal neutrophils conferring a clinical phenotype on female carriers as they age [46].

Laboratory diagnosis of CGD can be achieved by performing flow cytometric analysis to evaluate NADPH oxidase activity (oxidative burst) using dihydrorhodamine (DHR) 1,2,3 as a fluorescent marker of hydrogen peroxide generation. This is a relatively rapid and highly sensitive assay and allows the use of whole blood without purification of neutrophils, and is reasonably stable allowing measurements to be performed up to 48 hours after blood collection. Due to these reasons, this assay has replaced superoxide measurements and the Nitroblue tetrazolium (NBT) slide test as the primary screening assay for CGD [46,54-56].

Genetic testing is used for identification of the specific gene (encoding a subunit of NADPH oxidase) and relevant mutation. For the majority of CGD cases, gene sequencing of the *CYBB *gene permits identification of the causal mutation. The majority of mutations (~70%) in this gene are single nucleotide changes, which include splice-site, nonsense and missense mutations, while the remaining ~30% of mutations are deletions and/or insertions [57].

DHR-based flow cytometry can also be used to identify patients with AR-CGD (Figure 3G), though this can be trickier to interpret and requires a certain level of skill as well as a more quantitative reporting format, which includes both the frequency of neutrophils positive for oxidative burst after PMA stimulation and the intensity of fluorescence per cell (MFI) [55,58]. Since there are 4 genetic defects (*CYBA*, *NCF1*, *NCF2 *and *NCF4*) associated with AR-CGD, one would either have to do mutation analysis for all four genes, which could be cost-prohibitive, or do additional second-tier screening tests, such as intracellular flow cytometry for the various subunits - p22phox, p47phox and p67phox [58] or immunoblot analysis prior to genetic testing. These are not widely available in clinical labs and are probably most often done in the research setting, which may, by default, necessitate genetic testing to identify the specific gene defect.

Flow cytometry can also be used for carrier detection for XL-CGD, which should typically reveal a mosaic pattern for DHR fluorescence. However, it should be kept in mind that the nature of random X-chromosome inactivation could result in either a near-normal or a highly abnormal pattern in the flow analysis for oxidative burst in female carriers. Therefore, genetic testing remains the most robust way to perform carrier identification, especially if the familial disease-causing mutation is known. The flow-based DHR test is not sensitive enough to identify obligate carriers (parents of patients) or sibling carriers of AR-CGD caused by *NCF1 *or *NCF 2 *mutations as there appears to be normal oxidative burst on stimulation of neutrophils (Figure 3H), and the assay has not been tested for *CYBA *carriers. Therefore, detection of AR-CGD carriers is best performed by genetic testing, though this can pose challenges with regard to the *NCF1 *gene, since several unrelated patients have been reported to have a dinucleotide deletion (ΔGT) in exon 2 of this gene [59-62]. A recombination event between the functional *NCF1 *gene and two pseudogenes, on the same chromosome, carrying this ΔGT leads to the incorporation of the deletion into the *NCF1 *gene. This phenomenon renders carrier testing for p47phox defects difficult because normal individuals are apparently heterozygous for this GT deletion due to the pseudogenes. There are potential solutions to this problem [63,64], and while normals can be distinguished from patients and carriers, it remains unknown whether the "hybrid' protein expressing part of the sequence from the *NCF1 *gene with part of the sequence from the pseudogenes is really functional [65], and therefore, only *NCF1*-defective patients have been identified so far.

Prenatal diagnosis for CGD can be performed by fetal DNA testing along with gender analysis, if the familial mutation is known, from a chorionic villus sample (CVS) or amniotic fluid cells. The gene sequence from the fetus should be compared to the mother and a symptomatic family member as well as a normal individual to determine to confirm and validate the result. A combination of flow cytometric DHR analysis, genetic testing and family history was useful and relevant in the diagnosis of these two patients with CGD.

As the above cases exemplify, the diagnostic approach for most primary immunodeficiencies include a variety of laboratory tests and techniques, and several, but not all, of these analyses (Table 3) can be performed by multicolor and/or multiparametric flow cytometry [2,3]. In the case of monogenic defects, genetic testing remains the most valuable test for confirming a diagnosis, providing specific gene and mutation information as well as enabling genotype-phenotype correlations [5,6]. The organization and characterization of mutations for specific PID-related genes has become streamlined and widely available through the primary immunodeficiency databases [66] enabling correlation of new and previously identified mutations with clinical and immunological phenotype, besides family information.

**Table 3:** Non-disease-specific immunological tests used for the diagnosis of PIDs

Immunological Tests	Method(s)
Complete blood count (CBC) with differential	Automated hematology analyzer
Immunoglobulin quantitation - IgG, IgA, IgM, IgD, IgE	Immunoassay methods*
IgG, IgA subclass quantitation	Immunoassays
Lymphocyte subset quantitation - T, B and NK cells	Flow cytometry (FC)
B cell subset immunophenotyping (naïve B cells, memory B cell subsets, transitional B cells, plasmablasts, CD21^low ^B cells)	FC
T cell subset immunophenotyping (T cell subsets - naïve, activated and memory, Th17 T cells, regulatory T cells)	FC
NK cell subset immunophenotyping (Cytotoxic and cytokine-producing NK cells, NKT cells, measurement of perforin, granzyme A, granzyme B, IFN-gamma, CD107a/CD107b for functional proteins)	FC
Complement pathways (classical, alternate, mannose-binding lectin)	Immunoassays, Hemolytic assays
Cytokines	In plasma or tissue culture, after T cell stimulation (multiplex methods - Luminex^® ^or flow cytometry), in cells by intracellular flow cytometry, ELISPOT
Soluble activation or inflammatory markers - e.g. soluble BAFF, soluble CD25 (IL-2R)	Immunoassays or multiplex flow cytometry
Antibody responses to vaccine antigens Diphtheria, tetanus, Pneumococcal, *Hemophilus influenzae *among others)	Serological methods, multiplex methods (e.g. Luminex^®^)
Lymphocyte proliferation (mitogens, antigens, anti-CD3 stimulation)	Thymidine (3H-t) method, FC (CFSE, Edu^®^)
Thymopoiesis (TREC, CD4/CD8 recent thymic emigrants)	Real-time PCR, FC
TCR receptor diversity	Spectratyping -molecular, FC
NK cytotoxicity (spontaneous killing, ADCC, IL-2-stimulated and PHA stimulated cytotoxicity)	Radioactive method, FC
CD8 T cell cytotoxicity - mitogen-stimulated, antigen-specific	Radioactive method, FC
Costimulatory molecules	FC
TLR signaling pathways and phosphorylated proteins	FC, specific cytokines after TLR stimulation, Immunoblot analysis
Mutation analysis for monogenic defects of immune components	DNA-based gene sequencing
Measurement of innate immune responses	FC
Chromosomal studies for chromosomal defects - deletion, translocations and rearrangements	Fluorescence in-situ hybridization (FISH), array comparative genomic hybridization (aCGH)
Antigen-specific T cell quantitation	Tetramers/Pentamers/Dextramers^® ^by FC
Adenosine deaminase (ADA), Purine nucleoside phosphorylase (PNP), Gluocse-6 phosphate dehydrogenase (G6PD), Myeloperoxidase (MPO)	Enzyme assays
Adhesion molecules for Leukocyte Adhesion deficiencies (CD18, CD11a, CD11b, CD15)	FC
Neutrophil oxidative burst^^^	DHR test by FC (Nitroblue tetrazolium -NBT- test can also be used)
Delayed type Hypersensitivity	*In vivo *skin test
Autoantibodies (for PID-associated autoimmunity or autoantibody-related cytopenias)	Direct antiglobulin test (DAT or Coombs' test) for autoimmune hemolytic anemia, Immunoassays

*For a detailed list of immunoassay methods (see Table 3, page 11, Chapter 3 - Protein Analysis for Diagnostic Applications, by AT Remaley and GL Hortin, In Molecular Clinical Laboratory Immunology, Eds, Detrick, Hamilton and Folds, 7^th ^Ed), ^^ ^Neutrophil chemotaxis and phagocytic cells have limited clinical utility, DHR - Dihydrorhodamine 1,2,3; a bone marrow biopsy can be performed for further evaluation of certain PIDs, e.g. abnormal retention of neutrophils in the marrow (myelokathexis in WHIM syndrome), aberrant production of hematopoietic precursors (Reticular dysgenesis and congenital neutropenias).

While the above examples showcase the utility of flow cytometry to evaluate specific protein defects in the diagnosis of PIDs, it is also a very versatile tool for immunophenotyping of lymphocyte subsets and assessing lymphocyte or other leukocyte subset functions in PIDs. For example, defects in circulating B cells have been recognized in the very heterogeneous PID -Common Variable Immunodeficiency (CVID) for a number of years, and over time, several classifications involving B cell subsets and immunophenotyping have evolved in an effort to organize and stratify this complex and multifaceted immunodeficiency [11,67-73]. Similarly, T cell immunophenotyping has been used to identify abnormalities or changes in naïve, memory, effector, activated, TH17 inflammatory T cells, regulatory T cells (CD4+CD25+FOXP3+) and recent thymic emigrant (RTE) populations for diagnosis of several combined or cellular immunodeficiencies such as severe combined immunodeficiency (SCID), Omenn syndrome, Hyper IgE syndrome (HIES), IPEX (immunodeficiency, polyendocrinopathy, enteropathy, X-linked), CVID and DiGeorge (chromosome 22q11.2 deletion) syndrome among others [74-90].

Heterogeneity in lymphocyte subsets is not restricted to only T and B cells, but also present in the NK cell compartment, and multicolor flow cytometry can be used to immunophenotype human NK cells in various PIDs where NK cell defects are either primary or secondary [91-95]. However, when performing immunophenotyping for circulating lymphocyte subsets, it must be kept in mind that to obtain analytically stringent data, various factors, such as diurnal changes, acute exercise, hormonal alterations, age and gender influence these populations, quantitatively and qualitatively (especially relevant for serial monitoring), and this must be taken into consideration [96-100].

Diagnosis of PIDs with T cell defects also often involves the use of molecular techniques, besides flow cytometry, and these include analysis of thymic function and T cell receptor repertoire diversity [101]. Quantitation of T cell receptor excision circles (TREC), which are episomal by-products of T cell receptor rearrangement, by polymerase chain reaction (PCR) methods, especially real-time PCR, has been used to determine thymic output [102-104]. However, it should be kept in mind that TREC levels are affected by cellular division as well as the longevity of naïve T cells in the periphery [102] and therefore, may not be always useful as a marker for **recent**thymic emigration. But, use of TREC in conjunction with quantitative analysis of naïve T cells and/or recent thymic emigrants (RTE) by flow cytometry [83,86]is likely to provide a comprehensive assessment of thymic function. Accurate interpretation of TREC and RTE data requires correlation with total T cell counts along with the use of age-appropriate reference values derived from healthy donors, both pediatric and adults (Hoeltzle et al, manuscript in preparation). T cell receptor (TCR) repertoire diversity can be assessed by flow cytometry, however since the panel of reagents available covers only 2/3^rd ^of the known TCR -beta gene -variable region (TCR Vβ) families, molecular techniques, such as immunoscope analysis (spectratyping), have been found to be more sensitive and stringent [105-109].

Besides identifying quantitative anomalies in various immune cell populations by flow cytometry, functional assessment of these cell populations is equally important and can be achieved, for the most part, by the same methodology, though other methods can also be used. For example, measurement of lymphocyte proliferation to mitogens, such as Phytohemagglutinin (PHA), Pokeweed mitogen (PWM) and Concanavalin A (Con A), and antigens, such as *Candida albicans *(CA) and Tetanus toxoid (TT) to ascertain T cell immune competence in PIDs [110] has long been performed by DNA incorporation of radiolabeled thymidine (^3^H-T) after stimulation of peripheral blood mononuclear cells (PBMCs) with the appropriate agent. Elimination of techniques involving radioactivity is always beneficial to the clinical laboratory, and flow cytometry-based methods, primarily using the intracellular fluorescent dye, CFSE (carboxyfluorescein diacetate succinimidyl ester), are now available for measuring cellular proliferation [111-113]. However, a recent study seems to suggest that the use of CFSE to measure lymphocyte proliferation for the diagnosis of cellular PIDs would be inaccurate due to the high rate of false positive results [114]. CFSE is also difficult to use in a high-throughput clinical laboratory due its light-sensitive nature and the requirement for pre-labeling of cells.

A more attractive alternative has been the direct incorporation into DNA of a non-radioactive compound, an alkyne-modified nucleoside (EdU, 5-ethynyl-2'deoxyuridine), which is fluorescently tagged through covalent interaction with a dye-labeled azide, and used to visualize cell proliferation by flow cytometry [115,116]; Erickson et al, manuscript in preparation). The flow cytometry method of measuring proliferation offers several distinct advantages over the radioactive method, besides the obvious elimination of radioactivity, including, the ability to measure cellular proliferation in distinct lymphocyte subsets, and assess cellular viability, apoptosis and death using appropriate markers, such as Annexin V and 7-AAD, in the same assay. Flow cytometry also allows measurement of other cellular functions, such as phosphorylation of proteins involved in cell signaling pathways [117,118], though these assays are typically available at present only in larger clinical reference or research laboratories. An example of protein phosphorylation key to immune regulation includes the JAK-STAT pathway [119,120], and mutations in at least three STAT family members (STAT1, STAT3, STAT5B) are known to be associated with distinct PIDs [121-126].

Laboratory evaluation is essential not only for the diagnosis of PIDs, but also for the evaluation and measurement of recovery of immune function after therapeutic intervention, especially, but not exclusively, in hematopoietic stem cell transplantation (HSCT) [127-133]. However, timely treatment requires early diagnosis, especially of PIDs that are fatal, if left untreated, such as SCID or severe T cell lymphopenia [134-136]. The adoption of newborn screening (NBS) for SCID and other T cell deficiencies as part of the NBS panel, by the federal advisory committee on heritable disorders in newborns and children, in 2010 has ushered in a new era of population-based screening for these critical PIDs. The screening protocol involves detection of TREC in dried blood spots, followed by additional confirmatory flow cytometry and genetic testing when appropriate [137-144]. Early identification of SCID and T cell-deficient patients through the NBS program will pave the way for these infants to receive rapid intervention resulting in improved overall survival.

In conclusion, laboratory-based testing for PIDs is a rapidly expanding, constantly evolving field that plays an integral role in the diagnostic work-up of these complex immunodeficiencies, but also simultaneously provides valuable insights into human immunobiology. However, quality control and standardization of techniques, methods, platforms and reference values is essential to successful and accurate outcomes for immunological analyses within the laboratory, and clinical trial models may provide a frame of reference for such endeavors [145,146].

## Competing interests

The author declares that they have no competing interests.

## Appendix

Detailed Figure Legends:

Figure 1A**. Flow cytometric evaluation for Btk protein in a healthy control**. Btk protein analysis is performed by intracellular flow cytometry in B cells and monocytes from a normal donor. The B cells (marked in blue) and the monocytes (olive) express normal levels of Btk protein intracellularly as would be expected (lower plot, right of solid line). The isotype control is shown in the top panel (left of solid line).

**1B. Flow cytometric evaluation for Btk protein in Case 1 patient**. Btk protein analysis is performed by intracellular flow cytometry in monocytes (since B cells were absent) from the patient. The monocytes (olive) express normal levels of Btk protein intracellularly (lower plot). However, the presence of protein does not eliminate the possibility of functional defects. The isotype control is shown in the top panel (left of solid line).

**1C. Full-gene sequencing in the *BTK *gene for mutation analysis in Case 1 patient**. Full-gene sequencing in the forward (F) and reverse (R) direction (all exons, intron-exon boundaries and 5' and 3' untranslated regions were covered) of the *BTK *gene in patient (A) and wild-type normal control (B) revealed the presence of a hemizygous nonsense mutation in exon 15 (old nomenclature, exon 14 -new nomenclature, g.68137 G>A; c.1895 G>A, TGG>TAG; p.W588X) resulting in premature truncation of the translated protein. Since the defect was present in the latter part of the C-terminal portion of the protein it allowed for normal protein expression within monocytes but abrogated function. Six other XLA patients, besides this patient, have been described as having this specific mutation in the *BTK *gene.

**1D. Schematic representation of Btk protein structural organization**. The Btk protein has several distinct domains and is a member of the Tec-family of kinases, which are non-receptor tyrosine kinases. The five domains of Btk include a pleckstrin-homology domain (PH), a Tec-homology domain (TH) and 3 Src-homology domains (SH). The nonsense mutation present in the patient was in the SH1 kinase (key functional region) domain resulting in a loss of 72 amino acids in the C-terminal portion of the protein.

**1E. Schematic representation of Btk in B cell development**. Btk plays a key role in B cell development in the bone marrow and partially contributes to the transition of pro-B cells to pre-B cells (dotted line) from the pro-B cell to pre-B cell stage, but is really crucial for differentiation of pre-B cells into immature B cells (represented by solid line). Absence of Btk protein leads to an arrest in B cell development and significant B cell lymphopenia in the periphery. Btk expression in the normal B cell lineage is downregulated in plasma cells.

Figure 2A**. Pedigree analysis for patient (Case 2) with X-linked thrombocytopenia (XLT)**. XLT is an allelic variant of Wiskott-Aldrich syndrome (WAS) and is due to mutations in the *WAS *gene.

**2B. Flow cytometric analysis for Wiskott-Aldrich syndrome protein (WASP) in lymphocytes in XLT patient and carrier**. Data shown in this figure is obtained from Kanegane et al (ref number 38). Intracellular flow cytometry was performed in lymphocytes from an XLT patient, carrier mother and healthy control. The patient shows partial expression of WASP consistent with the milder clinical and immunological phenotype observed in XLT patients. The carrier mother resembles the control with normal expression of WASP in lymphocytes.

**2C. Flow cytometric analysis for Wiskott-Aldrich syndrome protein (WASP) in lymphocytes in WAS patient**. Data shown in this figure is obtained from Kawai et al (ref number 21). Intracellular flow cytometry was performed in T, B and NK cells from a healthy control (top panel) and a WAS patient (lower panel). The patient depicted here shows no expression of WASP. Absence of protein correlates with a severe phenotype in WAS patients.

Figure 3A**. Flow cytometric analysis for neutrophil oxidative burst (NOXB) in a healthy control**. Neutrophils from a healthy donor are evaluated for NADPH oxidase activity before (unstimulated) or after stimulation with Phorbol Myristate Acetate (PMA). The principle of this assay is that a non-fluorescent compound, Dihydrorhodamine 1,2, 3 when phagocytosed by normal, activated neutrophils (post-PMA stimulation) is oxidized by hydrogen peroxide produced during normal activated neutrophil respiratory burst, to Rhodamine 1,2,3, a green fluorescent compound, which can be detected by flow cytometry. Therefore, the fluorescence detected is an indirect measure of neutrophil oxidative burst function (Oxidized DHR1,2,3). The healthy control demonstrates normal neutrophil oxidative burst after stimulation.

**3B. Flow cytometric analysis for neutrophil oxidative burst (NOXB) in a patient with X-linked Chronic Granulomatous Disease (XL-CGD), Case #3**. Absence of normal oxidative burst in the majority of neutrophils (96%) is observed in the patient sample after stimulation, similar to that seen in the unstimulated sample. There is a very small population (4%) of neutrophils which show oxidative burst after stimulation. This result is consistent with a diagnosis of XL-CGD.

**3C. Full-gene sequencing in the *CYBB *gene for mutation analysis in Case 3 patient**. Full-gene sequencing of the *CYBB *gene, encoding the gp91phox protein, in the patient (A), was performed in the forward (F) and reverse (R) direction (all exons, intron-exon boundaries were covered) and revealed the presence of a hemizygous nonsense mutation in exon 5, p.R130X (reference wild-type *CYBB *sequence provided in panel B). This was established as a *de novo *mutation in the patient since the mother was not a carrier for this specific mutation.

**3D. Flow cytometric analysis for neutrophil oxidative burst (NOXB) in mother of patient with X-linked Chronic Granulomatous Disease (XL-CGD), Case #3**. Normal oxidative burst in the majority of neutrophils is observed in the mother's sample after stimulation (see right of dotted and solid lines, not accounting for the modest background in the unstimulated sample), similar to that seen in a healthy control. The limited background activation observed in the unstimulated sample can be seen in samples due to time lapse from blood collection and transportation conditions (right of solid line marks oxidative burst accounting for the background). This result is therefore not consistent with carrier status for XL-CGD, which was verified by gene sequencing (data not shown).

**3E. Schematic representation of NADPH oxidase**. NADPH oxidase, a key enzyme in the respiratory burst pathway consists of 5 subunits, 2 of which are membrane-bound - gp91phox and p22phox. The remaining 3 cytosolic subunits include the p47phox, p67phox and p40phox. These interact with Rac1, a RacGTPase molecule. Mutations in *CYBB *resulting in defective gp91phox account for the majority of cases of Chronic Granulomatous Disease (CGD).

**3F. Flow cytometric analysis for neutrophil oxidative burst (NOXB) in a carrier with X-linked Chronic Granulomatous Disease (XL-CGD), Case #4**. Two populations are observed for neutrophil oxidative burst after stimulation - a larger negative and a smaller positive population, consistent with carrier status for XL-CGD, which was confirmed by gene sequencing (heterozygous 16 bp deletion in the *CYBB *gene, c.360-375del16) and family history. The patient was clinically symptomatic for CGD consistent with the skewing of lyonization (X-chromosome inactivation) observed in the flow cytometry assay for neutrophil oxidative burst.

**3G. Flow cytometric analysis for neutrophil oxidative burst (NOXB) in a patient with autosomal recessive CGD (AR-CGD)**. Neutrophils from a female patient shows impaired oxidative burst after stimulation in a pattern consistent with AR-CGD. Flow analysis was confirmed by gene sequencing which revealed a mutation in the *NCF1 *gene encoding p47phox, which accounts for the majority of AR-CGD cases.

**3H. Flow cytometric analysis for neutrophil oxidative burst (NOXB) in a carrier with autosomal recessive CGD (AR-CGD)**. Normal neutrophil oxidative burst observed after stimulation in female patient, similar to healthy control. Family history revealed a female sibling diagnosed with AR-CGD with a pathogenic mutation identified in the *NCF1 *(p47phox) gene. However, the flow cytometry assay cannot be effectively used to identify carriers for AR-CGD.
